# Chromogranin A as Serum Marker for Gastroenteropancreatic Neuroendocrine Tumors: A Single Center Experience and Literature Review

**DOI:** 10.3390/cancers4010141

**Published:** 2012-02-15

**Authors:** Svenja Nölting, Axel Kuttner, Michael Lauseker, Michael Vogeser, Alexander Haug, Karin A. Herrmann, Johannes N. Hoffmann, Christine Spitzweg, Burkhard Göke, Christoph J. Auernhammer

**Affiliations:** 1 Department of Internal Medicine II, Campus Grosshadern, University-Hospital of the Ludwig-Maximilians-University of Munich, Munich 81377, Germany; E-Mails: svenja.noelting@med.uni-muenchen.de (S.N.); a.kuttner@gmx.net (A.K.); christine.spitzweg@med.uni-muenchen.de (C.S.); burkhard.goeke@med.uni-muenchen.de (B.G.); 2 Institute of Medical Informatics, Biometry and Epidemiology, University of Munich, Munich 81377, Germany; E-Mail: lauseker@ibe.med.uni-muenchen.de; 3 Department of Clinical Chemistry, Campus Grosshadern, University-Hospital of the Ludwig-Maximilian-University of Munich, Munich 81377, Germany; E-Mail: michael.vogeser@med.uni-muenchen.de; 4 Clinic of Nuclear Medicine, Campus Grosshadern, University-Hospital of the Ludwig-Maximilian-University of Munich, Munich 81377, Germany; E-Mail: alexander.haug@med.uni-muenchen.de; 5 Institute of Radiology, Campus Grosshadern, University-Hospital of the Ludwig-Maximilian-University of Munich, Munich 81377, Germany; E-Mail: karin.herrmann@med.uni-muenchen.de; 6 Department of Surgery, Campus Grosshadern, University-Hospital of the Ludwig-Maximilians-University of Munich, Munich 81377, Germany; E-Mail: johannes.hoffmann@med.uni-muenchen.de

**Keywords:** neuroendocrine tumors, gastroenteropancreatic system, sensitivity, chromogranin A, CIS-bio assay, alkaline phosphatase, urinary 5-hydroxyindoleacetic acid, liver metastases

## Abstract

The aim of this study was to assess the clinical sensitivities of the tumor markers chromogranin A (CgA), urinary 5-hydroxyindoleacetic acid (5-HIAA) and alkaline phosphatase (AP) in neuroendocrine tumors (NETs) of the GastroEnteroPancreatic-(GEP-) system depending on tumor primary location and metastatic spread. In a retrospective single-center series, sensitivities were evaluated in serum samples from 110 patients with midgut (n = 62) and pancreatic (n = 48) NETs. CgA levels were analyzed by a commercially-available immunoradiometric assay (CIS-bio) during routine follow-up in the years 2000–2009. CgA showed a higher sensitivity for midgut (68%) than pancreatic (54%) NETs. A higher CgA sensitivity and significantly higher median CgA values were found in patients with liver metastases than in those without, and in patients with hepatic and additionally extra-hepatic metastases than in those with hepatic and nodal metastases alone, respectively. We found an overall sensitivity for elevated 5HIAA excretion of 69% for midgut NETs and a significant correlation between median CgA and 5-HIAA values. The sensitivity of AP and the correlations of AP/CgA-data-pairs were low in both midgut and pancreatic NETs, although highest for metastatic pancreatic NETs. The sensitivity of CgA measurement depends on the NET primary location and spread of disease. 5-HIAA and CgA showed comparable sensitivity in midgut NETs, while AP does not seem to be useful as a tumor marker in GEP-NETs.

## 1. Introduction

Chromogranin A (CgA) is an acidic glycoprotein which is exclusively expressed in the secretory dense core granules of most normal and neoplastic neuroendocrine cell types [1]. It is co-released with peptide hormones [2,3]. Elevated circulating CgA levels have been demonstrated in serum or plasma of patients with various hormone-secreting or non-hormone secreting neuroendocrine tumors (NETs) [4,5,6,7]. Therefore, CgA is widely used and is recommended by most societies (ENETS, UKINETS, NANETS) as a general serum marker for NETs [2,5,6,8,9,10,11,12,13]. The clinical sensitivity of CgA has been demonstrated to depend on the assay utilized for serum or plasma CgA determination [14,15,16], on the threshold cut-off [15,16], on NET primary location [1,17,18], and on the spread of the disease, especially the existence of liver metastases [5,16,19,20]. High CgA levels correlate with tumor burden and are considered as a predictor of bad prognosis in both midgut and pancreatic NETs [5,19,20,21,22,23,24].

Clancy *et al*. suggested that alkaline phosphatase (AP) might be superior to CgA in predicting the survival of patients with NETs: serum AP levels above normal were suggested to correlate with a shorter survival of these patients [25]. We have therefore analyzed our single-center experience with CgA as tumor marker. Our aim was to assess the sensitivity of CgA depending on tumor primary location and the existence of liver and additional extra-hepatic metastases. We further investigated the sensitivity of AP in midgut and pancreatic NET patients depending on the presence of liver metastases, and examined if there was a significant correlation between serum CgA levels and serum AP levels in pancreatic and midgut NET patients. As Korse *et al*. postulated that serum CgA was superior to urinary 5-HIAA concerning the prognostic relevance in follow-up of metastatic midgut NETs [24], we also determined the sensitivity of 5-HIAA in midgut NET patients depending on the presence of liver metastases, and examined whether there was a significant correlation between median serum CgA levels and median urinary 5-HIAA levels.

## 2. Results and Discussion

### 2.1. Chromogranin A (CgA)

Sixty-eight of the 110 patients of the study population showed elevated median CgA levels; 26 of the 48 patients with a pancreatic NET and 42 of the 62 patients with a midgut NET revealed elevated median CgA levels. Table 1 shows the overall sensitivity in our study, population depending on different cut-off levels.

**Table 1:** Sensitivities of CgA depending on different cutoff levels in our study population (CIS-bio IRMA kit).

CIS-Bio IRMA Kit	Sensitivity	CgA Cut-Off Level (ng/mL)
n = 110	62%	<98
n = 110	73%	<70
n = 110	83%	<53

n: number of patients.

We examined differences in CgA sensitivities between pancreatic and midgut NET patients (Table 2).

**Table 2:** CgA sensitivities (number of patients with median CgA values above reference range/number of all patients of the corresponding group) depending on tumor primary.

CIS-Bio IRMA Kit	Pancreatic NETs	Sensitivity in Pancreatic NETs	Midgut NETs / Carcinoids	Sensitivity in Midgut NETs / Carcinoids
CgA Cutoff level (ng/mL): 98	n = 48	26/48 (54%)	n = 62	42/62 (68%)

n: number of patients.

Moreover, we compared CgA sensitivities between NET patients with liver metastases and those without (Table 3), and additionally, between NET patients with localized disease, those with lymph node metastases only, those with liver (and lymph node) metastases only, and those with additional extra-hepatic metastases (Table 4).

**Table 3:** CgA sensitivities (number of patients with median CgA values above reference range/number of all patients of the corresponding group) depending on hepatic metastatic spread and tumor primary.

Sensitivity of CgA *	No Liver Metastases	Liver Metastases
**Pancreatic NETs (n = 48)**	4/13 (31%)	22/35 (63%)
**Midgut NETs (n = 62)**	6/15 (40%)	36/47 (77%)
**Total**	10/28 (36%)	58/82 (71%)

* CgA Cutoff level (ng/mL): 98; n= number of patients.

**Table 4:** CgA sensitivities (number of patients with median CgA values above reference range/number of all patients of the corresponding group) depending on metastatic spread and tumor primary.

Sensitivity of CgA *	Localized Disease	Lymph Node Metastases Only	Pulmonary Metastases Only	Lymph Node and Pulmonary Metastases Only	Liver (± Lymph Node) Metastases Only	Liver Metastases and Additional Bone, Peritoneal or Pulmonary Metastases
**Pancreatic NETs (n = 48)**	3/10 (30%)	1/2 (50%)	0/1 (0%)		15/25 (60%)	7/10 (70%)
**Midgut NETs (n = 62)**	3/9 (33%)	2/5 (40%)		1/1 (100%)	20/28 (71%)	16/19 (84%)
**Total**	6/19 (32%)	3/7 (43%)	0/1 (0%)	1/1 (100%)	35/53 (66%)	23/29 (79%)

* CgA Cutoff level (ng/mL): 98; n = number of patients.

**Figure 1:**
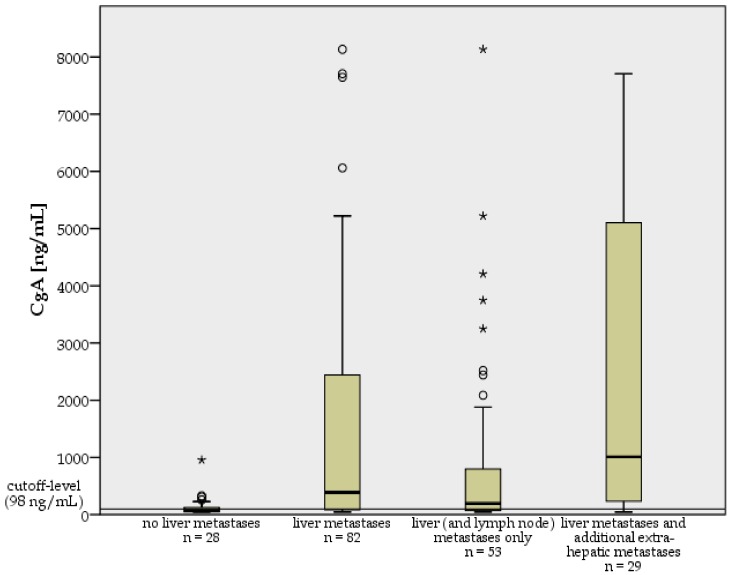
Box plots of median serum CgA levels (CgA in ng/mL) for the investigated 110 pancreatic and midgut NET patients, subdivided into patients without and patients with liver metastases, patients with liver (and lymph node) metastases only and patients with hepatic and additional extra-hepatic metastatic spread; n = number of patients.

**Figure 2:**
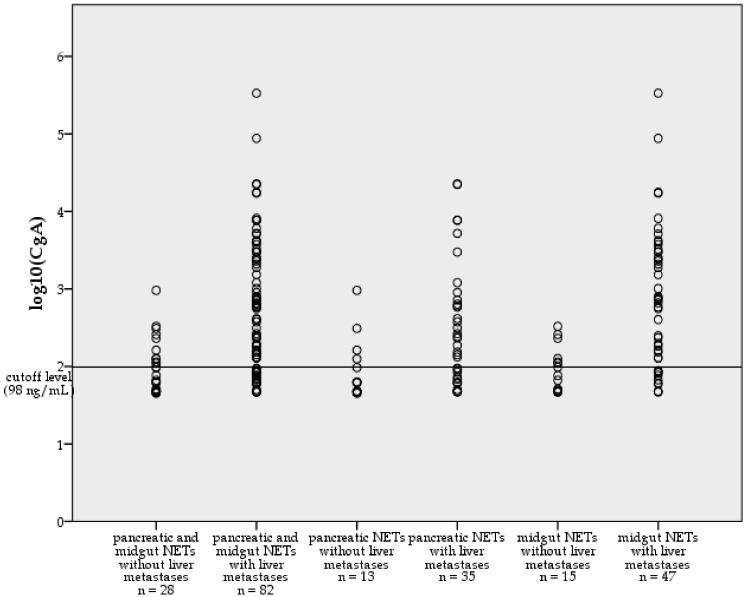
Log_10_-transformed median serum CgA levels [log10(CgA), CgA in ng/mL] of the whole study population (n = 110), pancreatic NET patients (n = 48) and midgut NET patients (n = 62), subdivided into patients without liver metastases and patients with liver metastases. n = number of patients.

In addition, we compared the median CgA values between NET patients with liver metastases and those without, and between NET patients with liver (and lymph node) metastases only, and those with additional extra-hepatic metastases (Figure 1 and Figure 2).

NET patients affected by liver metastases showed significantly higher median CgA values (n = 82; median ± standard deviation: 389 ± 38,103 ng/mL; range: 47–335,000 ng/mL) than those without liver metastases (n = 28; median ± standard deviation: 65 ± 181 ng/mL; range: 45–957 ng/mL) by Mann-Whitney-Test (*p* < 0.0001) (Figure 1 and Figure 2). Moreover, significantly higher median CgA values were found in patients with hepatic and additional extra-hepatic metastatic spread (n = 29; median ± standard deviation: 1,011 ± 63,224 ng/mL; range: 48–335,000 ng/mL) compared to patients with liver (and lymph node) metastases only (n = 53; median ± standard deviation: 196 ± 4,427 ng/mL; range: 47–22,642 ng/mL) (*p* = 0.005) (Figure 1). Additionally, we compared median CgA values above the reference range (cutoff level: 98 ng/mL) in pancreatic NET patients without liver metastases (n = 4; median ± standard deviation: 236 ± 387 ng/mL; range: 125 ng/mL–925 ng/mL) to median CgA values above reference range in pancreatic NET patients with liver metastases (n = 22; median ± standard deviation: 593 ± 6,573 ng/mL; range: 132 ng/mL–22,642 ng/mL) (Figure 2). The difference between these two subgroups was not significant by Mann-Whitney-Test (*p* = 0.177). In 22 pancreatic NET patients with liver metastases and elevated median CgA values, the range of all single CgA measurements (n = 132) was 98–161,000 ng/mL and the median of all single CgA measurements was 541 ± 18,458 ng/mL. In 4 pancreatic NET patients without liver metastases and elevated median CgA values, the range of single CgA measurements (n = 20) was 125 to 4,242 ng/mL, and the median of all single CgA values was 368 ± 992 ng/mL. In the subgroup of pancreatic NET patients we further compared median CgA values of patients with liver ± lymph node metastases only (n = 25, median ± standard deviation: 153 ± 6,171 ng/mL; range: 47–22,642 ng/mL) to median CgA values of patients with hepatic and additional extra-hepatic metastases (n = 10, median ± standard deviation: 498 ± 3,089 ng/mL; range: 48–7,707 ng/mL) and found no significant difference (*p* = 0.273). In contrast, midgut NET patients with liver metastases and elevated CgA levels (n = 36; median ± standard deviation: 1,704 ± 56,894 ng/mL; range: 128–335,000 ng/mL) showed significantly higher median CgA levels than those without liver metastases and elevated CgA levels (n = 6; median ± standard deviation: 178 ± 91 ng/mL; range: 110–329 ng/mL) (*p* = 0.002) (Figure 2). In 36 midgut NET patients with liver metastases and elevated median CgA values, the range of all single CgA measurements (n = 300) was 100–1,200,000 ng/mL and the median of all single CgA measurements was 1,521 ± 101,068 ng/mL. In six midgut NET patients without liver metastases and elevated median CgA values, the range of all single CgA measurements (n = 35) was 103–764 ng/mL and the median of all single CgA values was 259 ± 189 ng/mL. In addition, significantly higher median CgA levels were found in midgut NET patients with additional extra-hepatic metastatic spread (n = 19; median ± standard deviation: 2,293 ± 77,538 ng/mL; range: 83–335,000 ng/mL) than in those with liver ± lymph node metastases only (n = 28; median ± standard deviation: 325 ± 1,836 ng/mL; range: 47–8,133 ng/mL) (*p* = 0.012).

### 2.2. Alkaline Phosphatase (AP)

Elevated AP levels were found in 36 of the 110 patients of the study population, resulting in a low overall sensitivity of 33%; 20 of the 48 patients with a pancreatic NET and only 16 of the 62 patients with a midgut NET showed elevated AP levels. The sensitivities of AP depending on tumor primary and hepatic metastatic spread are shown in Table 5.

**Table 5:** Sensitivities of AP (number of patients with median AP values above reference range/number of all patients of the corresponding group) depending on tumor primary and hepatic metastatic spread.

Sensitivity of AP *	No Liver Metastases	Liver Metastases	Total
**Pancreatic NETs (n = 48)**	3/13 (23%)	17/35 (49%)	20/48 (42%)
**Midgut NETs (n = 62)**	1/15 (7%)	15/47 (32%)	16/62 (26%)
**Total**	4/28 (14%)	32/82 (39%)	36/110 (33%)

* AP Cutoff level (U/L): 135; n = number of patients.

In addition, we assessed the correlation coefficient between AP/CgA data-pairs, *i.e*., between every available serum CgA value and serum AP value determined at the same time in each individual. AP values and CgA values were reported as the ratio to the upper limit of normal. CgA/AP-data-pairs showed a low correlation in the midgut NET group (r = 0.208) and in the subgroup of patients with midgut NETs with hepatic metastases (r = 0.162). A slightly higher, but non-significant correlation was found between CgA and AP in the pancreatic NET group (r = 0.362) and in the subgroup of patients with pancreatic NETs with hepatic metastases (r = 0.432).

### 2.3. Urinary 5-Hydroxyindoleacetic Acid (5-HIAA)

Measurements of 24 h urinary 5-HIAA excretions were available in 51 of 62 midgut NET patients: 35 of the 51 midgut NET patients with available 24 h urinary 5-HIAA values showed elevated median 5-HIAA-levels (cutoff level: <9 mg/24 h), resulting in a sensitivity of 69%. Table 6 shows the sensitivities of urinary 5-HIAA depending on hepatic metastatic spread.

**Table 6:** Sensitivities of 5-HIAA (number of patients with median AP values above reference range/number of all patients of the corresponding group) depending on hepatic metastatic spread.

Sensitivity of 5-HIAA *	No Liver Metastases	Liver Metastases
**Midgut NETs (n = 51)**	0/11 (0%)	35/40 (88%)

* 5-HIAA Cutoff level (mg/24 h): <9; n: number of patients.

We detected significantly higher median 5-HIAA levels in midgut NET patients with liver metastases than in those without liver metastases (*p* < 0.0001). We found a significant correlation between median CgA values and median 5-HIAA values in patients with midgut NETs (Spearman correlation coefficient r = 0.752, *p* < 0.0001). We also detected a significant correlation between median CgA values and median 5-HIAA values in the subgroup of midgut NET patients with liver metastases (Spearman correlation coefficient r = 0.696, *p* < 0.0001), but not in the subgroup of midgut NET patients without liver metastases (Spearman correlation coefficient r = 0.036, *p* = 0.915).

### 2.4. Discussion and Literature Review

As shown in this study and previous studies, the sensitivity and specificity of CgA as tumor marker in neuroendocrine tumors depends on the cutoff level (Table 1 and Table 7) [14,15,16,26,27]. Table 7 summarizes the study populations (n), CgA sensitivities and specificities reported for the CIS-bio IRMA kit using different cutoff-levels in different studies [14,15,16].

Using a lower cutoff level for the assay causes a higher sensitivity but a lower specificity, and vice versa (Table 1 and Table 7). For routine diagnostic, we used approximately the same CgA cutoff-level (98 ng/mL) as used by Stridsberg *et al*. (99 ng/mL) [15], resulting in a comparable sensitivity. Higher sensitivities which we observed using lower cutoff-levels of 53 ng/mL and 70 ng/mL, respectively, approximately correlated with the sensitivities reported by Zatelli *et al*. [16] (cutoff: CgA < 53 ng/mL) and Ferrari *et al*. [14] (cutoff: CgA < 70 ng/mL), respectively. The DAKO ELISA kit demonstrated in most studies a higher sensitivity and specificity than the CIS-bio IRMA kit (Table 8) [14,15,16].

Stridsberg *et al*. suggested that the best compromise between sensitivity and specificity was with the use of the Eurodiagnostica radioimmunoassay (Table 9) [15].

CgA levels and sensitivities strongly depend on tumor primary location and may vary between different tumor entities [17], as has also recently been shown in a total of 1,721 patients from different studies by Modlin *et al*. [1]. Table 10 summarizes several studies sub-grouping patients according to tumor primary location, mostly reporting a tendency towards a higher CgA sensitivity in midgut NETs than for pancreatic NETs [4,14,15,20]. This finding is confirmed by our current study.

**Table 7:** Sensitivities and specificities of CgA (CIS-bio IRMA kit) in different studies.

CIS-bio IRMA Kit	Stridsberg *et al.* (n = 45)	Ferrari *et al.* (n = 93)	Zatelli *et al.* (n = 202)
**Sensitivity**	67%	79%	78%
**Specificity**	96%	64%	71%
**CgA Cutoff Level (ng/mL)**	<99	<70	<53

n = number of patients.

**Table 8:** Sensitivities and specificities of CgA depending on different cutoff levels (DAKO ELISA kit).

DAKO ELISA Kit	Stridsberg *et al.* (n = 45)	Ferrari *et al.* (n = 93)	Zatelli *et al.* (n = 202)
**Sensitivity**	85%	79%	84%
**Specificity**	85%	91%	85%
**CgA Cutoff Level [U/L]**	<19	<34	<16

n: number of patients.

**Table 9:** Sensitivity and specificity of CgA (Eurodiagnostica RIA assay).

Eurodiagnostica RIA	Stridsberg *et al*. (n = 45)
**Sensitivity**	93%
**Specificity**	85%
**CgA Cutoff Level (mmol/L)**	<4

n: number of patients.

Modlin *et al*. also reported a higher CgA sensitivity and higher CgA levels in ileal NETs than in pancreatic NETs [1]. In contrast, Schürmann *et al*. found highest CgA values for pancreatic NETs and slightly lower values for ileal NETs [18]. Interestingly, among the small subgroup of patients without liver metastases and elevated CgA levels, we observed higher median CgA values in patients with pancreatic NETs than in those with midgut NETs (236 ng/mL *vs*. 178 ng/mL, respectively), while in contrast, among patients with liver metastases and elevated CgA levels, higher median CgA values were found in patients with midgut NETs than in those with pancreatic NETs (1,704 ng/mL *vs*. 593 ng/mL, respectively). Arnold *et al*. assessed CgA levels in 344 patients with foregut (pancreas, duodenum, bronchus), midgut and hindgut NETs with a DAKO ELISA kit. They found highest CgA levels in patients with functioning midgut NETs (carcinoid syndrome) being accompanied by liver metastases, but higher CgA levels in pancreatic NETs than in non-functioning midgut NETs, consistent with our data [21]. In midgut NETs we found a significant difference between elevated CgA levels of patients with liver metastases and those of patients without. In contrast, in pancreatic NETs no significant difference was found between these two subgroups. For midgut NET patients our data provide better differentiation between non-hepatic metastasized, hepatic metastasized and additional extra-hepatic metastasized disease by CgA determination than for pancreatic NET patients. In contrast, Zatelli *et al*. assessed a higher positive predictive value (PPV) and a higher negative predictive value (NPV) for pancreatic NETs (84% and 78%) than for midgut NETs (40% and 46%) using the CIS-bio IRMA kit [16].

**Table 10:** CgA sensitivities in NETs classified in pancreatic and midgut NETs depending on tumor primary and assay (CIS-bio IRMA kit, DAKO ELISA kit, in-house RIA).

	Pancreatic NETs	Sensitivity in Pancreatic NETs	Midgut NETs / Carcinoids	Sensitivity in Midgut NETs / Carcinoids
Current Study ^1 ^ CgA Cutoff level (ng/mL): 98	n = 48 *	54%	n = 62	68%
Stridsberg *et al*. ^1 ^ CgA Cutoff level (ng/mL): 99	n = 16 *	69%	n = 11	73%
Ferrari *et al*. ^1 ^ CgA Cutoff level (ng/mL): 70	n = 36 *	58%	n = 14	85%
Tomassetti *et al*. ^2^ CgA Cutoff level (U/L): 17	n = 29 **	55%	n = 34	59%
Stridsberg *et al*. ^2 ^ CgA Cutoff level (U/L): 19	n = 16 *	81%	n = 11	91%
Ferrari *et al*. ^2 ^ CgA Cutoff level (U/L): 34	n = 36 *	92%	n = 14	81%
Nobels *et al*. ^3 ^ CgA Cutoff level (ng/mL): 175	n = 34 ***	32%	n = 59	80%

*: Pancreatic NETs; **: 21 nonfunctioning pancreatic NETs, 4 gastrinomas, 2 somatostatinomas, 2 Glucagonomas; ***: 13 pancreatic islet cell tumors, 21 insulinomas; n: number of patients; ^1^: CIS-bio IRMA kit; ^2^: DAKO ELISA Kit; ^3^: In-House RIA.

Moreover CgA levels and CgA sensitivities crucially depend on metastatic spread. Arnold *et al*. [21] found that the hepatic tumor burden significantly increased CgA levels. Additional extra-hepatic lymph node metastases in the presence of liver metastases did not further enhance CgA levels. Janson *et al*. also detected significantly higher plasma CgA levels among patients with multiple (≥5) liver metastases than in those with only few (≤5) liver metastases, or lymph node metastases alone [22]. Walter *et al*. found CgA levels to be significantly more elevated in metastatic NETs than in those with localized disease (74% *vs*. 51%) [28]. Campana *et al*. observed significantly higher CgA levels in patients with diffuse disease compared with patients with local or hepatic disease [26]. Zatelli *et al*. also reported significantly higher mean CgA levels in NET patients with metastases than in those without and observed higher CgA levels in patients with liver metastases than in those with locally advanced disease. Interestingly, by utilizing both a CIS-bio IRMA kit and a DAKO ELISA kit, Zatelli *et al*. found lower CgA levels in patients with extensive metastatic spread (extra-hepatic metastases) than in those with liver metastases only [16]. Consistent with the data reported by Arnold *et al*., Janson *et al*., Zatelli *et al*. and Walter *et al*., we also found significantly higher median CgA values and relevantly higher CgA sensitivities in NET patients with liver metastases than in those without (Figure 1 and Figure 2). Moreover, in our study lymph node metastases did not significantly increase CgA levels, the sensitivity being consistent with the findings by Arnold *et al*. and Janson *et al*. In contrast to the data reported by Zatelli *et al*., we report significantly higher median CgA values and higher CgA sensitivities in patients with hepatic and additional extra-hepatic metastatic spread than in patients with liver and lymph node metastases alone (*p* = 0.005) (Figure 1, Table 4), again confirming a strong dependence of CgA levels and CgA sensitivity on tumor burden. Only 31% of the pancreatic NET patients without liver metastases and 40% of the midgut NET patients without liver metastases showed median CgA values above the reference range in our study, indicating a poor overall sensitivity of CgA in patients without liver metastases. According to Zatelli *et al*., the DAKO ELISA kit showed a higher sensitivity and specificity for distinguishing between metastatic and non-metastatic NETs in first diagnosis or relapse than the CIS-bio IRMA kit [16].

Although CgA is currently the best available tumor marker indicating spread of disease, response to treatment [8,18,19,23], tumor recurrence [29,30] and poor prognosis [21,22,24,31], there are many limitations in its use, such as different co-morbidities and drugs that may increase CgA levels and lead to false positive results (summarized in Table 11, reviewed by Modlin *et al*. [1,32]).

**Table 11:** Factors interfering with CgA measurement.

	Falsely High Levels
**Carcinoma**	Hepatocellular carcinoma, pancreas carcinoma, colorectal cancer, small cell lung cancer, breast cancer, ovary cancer, prostate cancer, neuroblastoma
**Renal Disease**	Renal insufficiency
**Cardiovascular Diseases**	Arterial hypertension, cardiac insufficiency, acute coronary syndrome
**Gastrointestinal Disorders**	Chronic atrophic gastritis, pancreatitis, inflammatory bowel disease, irritable bowel syndrome, liver cirrhosis, chronic hepatitis
**Inflammatory Diseases**	Systemic rheumatoid arthritis, chronic bronchitis, airway obstruction in smokers
**Drugs**	PPI, H2RA
**Others**	Food intake and sports shortly before CgA measurement

None of these co-morbidities were considered in our retrospective study, thus limiting the data of this retrospective cohort. Further limitations due to the retrospective character of the study consist in the lack of information about tumor histopathologic classification, the proliferation index Ki-67 and tumor grading according to the WHO classification, as well as primary tumor staging according to the TNM classification, functioning or non-functioning characteristics and MEN1 status. These parameters have been reported to influence CgA levels, as reviewed by Modlin *et al*. [1]. Moreover, survival data were not considered, and therefore the prognostic impact of CgA was not addressed in this study. Furthermore, the retrospective character of the study did not allow us to assess false positive patients and therefore CgA specificity could not be determined.

A recent publication by Clancy *et al*. suggested that elevated AP levels in a multivariate analysis as robust adverse prognostic factor in patients with NETs [25]. We therefore examined the sensitivity of AP in our GEP-NET patients and explored whether there was a significant correlation between CgA and AP in midgut and pancreatic NET patients, respectively. The sensitivities of AP were low in the pancreatic and midgut NET group. Neither in pancreatic NETs nor in midgut NETs could we find a significant correlation between CgA and AP values. A considerably high number of AP/CgA-data-pairs, 24% in the pancreatic NET group and 43% in the midgut NET group, respectively, showed CgA levels above the reference range, but with normal AP levels. This was consistent with the results of Clancy *et al*. who found elevated AP levels in 46 (41%) of 113 patients, but elevated CgA levels in 78 (78%) of 100 patients (cutoff level: AP < 127 U/L; CgA < 39 ng/mL) [25]. According to our results, AP values are more often elevated in pancreatic NET patients than in midgut NET patients and higher in patients with advanced metastatic hepatic disease than in patients without liver metastases. The most likely reason for higher AP values associated with pancreatic NETs may be a compression or infiltration of the bile duct by the pancreatic tumor. Despite the fact that elevated serum AP might be a robust adverse prognostic factor, it cannot substitute for CgA as a tumor marker due to its low sensitivity.

Janson *et al*. reported a significant association between elevated 5-HIAA levels and a shorter survival in NET patients by univariate analysis [22], while Korse *et al*. observed no significant correlation between 5-HIAA levels and survival time [24]. We examined the sensitivity of 5-HIAA in midgut NETs and compared it to sensitivity of CgA in midgut NETs. We found approximately the same sensitivity of 5-HIAA (69%) and CgA (68%) in midgut NET patients. In the subgroup of midgut NET patients affected by liver metastases we demonstrated an even higher sensitivity for 5-HIAA (86%) than for CgA (77%). As expected, in the subgroup of patients without liver metastases, 5-HIAA was not elevated depending on the fact that hepatic metastatic spread is necessary to escape the first-pass effect of 5-HIAA. Therefore, determination of 5-HIAA is indicated in all midgut NETs where the carcinoid syndrome is suspected. The significant correlation found between median 5-HIAA and median CgA values was consistent with the results of Nobels *et al*. [5]. Unfortunately, in clinical practice there are a lot of limitations to 5-HIAA assessment, as many drugs, foods, natural stimulants and co-morbidities may alter the levels of 24h urinary 5-HIAA (summarized in Table 12).

**Table 12:** Disturbing factors of 5-HIAA measurement.

	Falsely High Levels	Falsely Low Levels
**Drugs**	Acetaminophen, cumarin, diazepam, fluorouracil, antihypertensive drugs, ephedrine, reserpine, mephenesin, phenobarbital, amphetamine	Heparin, aspirin, MAO-inhibitors, methyldopa, levodopa, tricyclic antidepressants, isoniacide, hydralazine
**Fruits**	Banana, pineapple, currant, plum, melon, gooseberry, mirabelles, kiwi	
**Vegetable**	Tomatoes, avocados, aubergines	
**Other Food**	Chocolate, walnuts	
**Natural Stimulants**	Nicotine, coffee, black tea	Alcohol
**Co-morbiditiesc**	Coeliac sprue, epilepsy	Renal insufficiency

## 3. Experimental Section

### 3.1. Patients and Laboratory Measurements

This was a single-center series of a total of 110 different patients (47 women and 63 men) with a histological diagnosis of a midgut NET or pancreatic NET between the years 2000 and 2009: 48 of the 110 NET patients had pancreatic NETs and 62 of them midgut NETs (jejunum/ileum).

The mean age of the total study population was 61 *±* 12 years (mean ± SD, range 30–88 years). In the pancreatic NET group the mean age was 58 *±* 11 years (range 31–88 years) and in the midgut NET group the mean age was 63 *±* 12 years (range 30–87 years). The test results received during clinical routine were retrospectively analyzed for CgA and AP serum levels as well as for 5-HIAA in 24 h-urine samples. In most cases multiple samples had been obtained from each subject on different occasions, resulting in a total of 678 measurements for CgA, 1,564 measurements for AP, and 188 measurements for 5-HIAA. For CgA determination a commercial immunoradiometric assay (IRMA; CGA-RIA CT, CIS-bio international-Shering, Gif-sur-Yvette, France) was used [inter-assay coefficient of variation (CV): 2.4% (mean concentration 172 ng/mL)]. Assay characteristics are described elsewhere [1]. If not separately mentioned, a CgA cutoff-level of 98 ng/mL was used. For AP determination a commercial photometric assay was utilized with a clinical chemical analyser [Olympus, Hamburg, Germany; inter-assay CV: 4.4% (mean concentration 55 U/L)] (cutoff level: <135 U/L). Urinary 5-HIAA concentrations were quantified by high performance-liquid chromatography with electrochemical detection (HPLC-ECD) utilizing a commercial assay kit [Chromsystems, Munich, Germany; inter-assay CV: 4.4% (mean concentration 3.1 mg/L)] (cutoff level: <9 mg/24 h). The quality assurance of all analyses was performed according to the Guidelines of the German Medical Association. A retrospective data analysis was performed in adherence with local regulations and approved by the local ethical committee.

### 3.2. Statistics and Data Analysis

We calculated the sensitivity of the different tumor markers by assessing the median of all collected values from each subject during follow-up, subsequently taking the percentage of all patients in the respective (sub-) group with median values above the reference range. The results are reported as medians. To compare different subgroups of patients, the Mann-Whitney test and Spearman rank correlations were used. Correlations of AP/CgA-data-pairs were calculated for the individual median values. Correlation coefficients within subjects were calculated as described in Bland and Altman [33]. *P* ≤ 0.05 was considered as significant, *p* ≤ 0.01 was considered as highly significant. Due to the explorative character of this work, all p-values have to be interpreted descriptively.

## 4. Conclusions

In summary, the sensitivity of CgA as a tumor marker in neuroendocine tumors of the GEP system depends on the threshold cut-off level [15,16,27,28], on the specific assay for serum or plasma CgA determination [14,15,16], on NET primary location [17,18] and on the spread of disease, especially the presence of liver metastases [5,16,19,20]. Our single-center experience confirms and extends previous results and suggests that CgA is especially of value for staging purposes in patients with disease with liver metastasis.
